# Is the Physician Authorised to Provoke Premature Artificial Abortion to Save the Mother?

**Published:** 1853-01

**Authors:** 


From the Jour. des Connais, Med. Chir.

*Is the Physician authorised to provoke Premature Artificial Abortion to save the Mother?*

A LONG discussion, in which some of the most distinguished medical savans have taken part, has been for some time going on before the *Academie de Medicine*, on the subject of artificial abortion. This controversy, for it has already reached that point, grew out of a report entitled, "*De l'accouchement premature artificial,*" presented to the Academy of Medicine on the 10th of February, 1852, by M. Cazeaux.

The following, as we think, just conclusion, closes the report of M. C. on the subject:

1st. It is in consequence of a false interpretation, that the laws, both human and divine, relative to abortion, have been applied to abortion practised with a conservative object.

2nd. Let the law punish crime; but they cannot reach, without injustice, an act accomplished with the purest intentions.

3d, Placed in the desperate alternative of choosing between the life of her infant and her own, the female has, by the laws of nature, the right to decide against her offspring.

4th. In this case the physician may, and should sacrifice the infant, for the safety of the mother.

5th, Provoked abortion being much less serious for the mother than embryotomy, performed at the full period of gestation, the accoucheur may, and ought to give it the preference.

6th. Deformities, in which the pelvis may be found less than six centimetres and a half in its shortest diameter—hemorrhages which nothing can check---tumors either in the hard or the soft parts, which cannot be removed---are the only indications which can call for provoked abortion.

7th. The physician should never decide upon a step of this serious nature without the previous advice of several enlightened medical men.

